# Non-Diarrheal Celiac Disease in Iraq: A Multicenter study

**DOI:** 10.34172/mejdd.2025.443

**Published:** 2025-10-31

**Authors:** Abdullah Zuhair Alyouzbaki, Ahmed Abdul Hussein Alhilly, Samar Saad Abdulhussein, Azhar Khalaf Alazawi, Kamal Breesam Lafta, Khalid N M Alkheroo

**Affiliations:** ^1^College of Medicine, University of Mosul, MRCP, CABM GI&H, Iraq; ^2^College of Medicine, Babylon University, FICMs GI & H, Iraq; ^3^College of Medicine, University of Kirkuk, FICMs GE&H, Iraq; ^4^Nursing Home Hospital / Gastroenterology and endoscopy Unit, FICMs GE&H, Iraq; ^5^GIT Basrah Hospital, FICMs GE&H, Iraq; ^6^College of Medicine, University of Mosul, CABMS Internal Medicine, FRCP, Iraq

**Keywords:** Celiac disease, Gluten sensitivity, Anemia

## Abstract

**Background:**

Non-diarrheal celiac disease (NDCD) is increasingly recognized in Iraq, but many cases are often missed or underdiagnosed because of its non-classical presentation. This study aimed to determine the clinical features and relevant investigations for patients with NDCD in Iraq.

**Methods:**

This is a prospective study that includes 117 cases of NDCD collected from different centers across Iraq. The study evaluated presenting symptoms, associated features, serological markers, endoscopic findings, and histopathological features. Patients with classical diarrhea were excluded from the study.

**Results:**

90 patients (77%) were women, and 94 patients (80%) were younger than 40 years. More than half had a normal body mass index, while a quarter of patients were classified as overweight and obese. Only 22% of patients with NDCD received a diagnosis within one year of the onset of symptoms, and 99 patients (84.6%) were suspected of having celiac disease during gastroscopy. Common symptoms among patients with NDCD included epigastric pain and anemia reported by 78.6% and 86.3% of patients, respectively. Endoscopic features suggestive of celiac disease were present in 94% of cases, and Marsh grade 3 histopathological features were observed in 75% of patients.

**Conclusion:**

NDCD is more prevalent in young, normal-weight, and obese female patients. Due to non-classical presentations such as epigastric pain or anemia, there is often a delay of more than one year in receiving a diagnosis. A gastroscopy performed by an experienced doctor yields a high diagnostic success rate in identifying NDCD. Furthermore, most patients with NDCD exhibit advanced histopathological features of celiac disease in their duodenal biopsies.

## Introduction

 Celiac disease (CD), also known as gluten-sensitive enteropathy, is a common chronic multi-organ immune-mediated inflammatory disorder of the small intestine that is triggered by sensitivity to dietary gluten and associated proteins in individuals with a genetic predisposition. Gluten comprises both prolamins and glutenins present in various types of wheat, rye, and barley, and is responsible for antigenicity and damage to the small intestinal mucosa. Grains that do not cause CD include rice, corn, and millet.^1^

 It affects approximately 1% of the global population. In most nations, a prevalence of 1:70 to 1:300 has been reported by epidemiological investigations using serological testing with biopsy verification.^2,3^

 Studies in the general population have revealed that diagnosed instances of CD may merely be the tip of the iceberg. For instance, an Italian study found that the ratio of asymptomatic to symptomatic patients was 7:1.^4^

 More than 95% of people with CD have HLA DR3-DQ2 and/or DR4-DQ8, compared with 30-40% of the general population in most countries.^5,6^ Type 1 diabetes mellitus and autoimmune thyroid disease are two autoimmune diseases linked to CD.^7^

 The risk is higher for first- and second-degree relatives of patients with CD. A meta-analysis found that the combined prevalence of CD was 8.9% (1:11) for siblings, 7.9% (1:13) for children, and 3% (1:33) for parents.^8^

 Different terms are used to describe different situations of CD like patients with classical CD are those who present with chronic diarrhea with symptoms or signs of malabsorption. Patients with atypical CD are those who lack prominent diarrhea or malabsorption and present with subtle gastrointestinal symptoms and extraintestinal manifestations like iron deficiency anemia, osteoporosis, infertility, elevated liver enzymes, and association with other autoimmune disorders. Subclinical CD is defined as those who have no symptoms and are diagnosed after presenting with upper gastrointestinal symptoms for other indications or by doing serological tests for high-risk groups, like those with a first-degree relative of the index case or patients with type 1 diabetes mellitus (DM). The term non-diarrheal celiac disease (NDCD) includes both atypical CD and subclinical CD.

 The peak age in adults is regarded as the third and fourth decades of life; nevertheless, it may also occur in the sixth decade of life. Consequently, the striking clinical picture of a child with life-threatening malabsorption is frequently supplanted by the largely atypical appearance of adult CD.^9^

 In adults, the presentations of CD are variable, and patients may present with gastrointestinal manifestations, including chronic diarrhea, steatorrhea, flatulence, and the consequences of malabsorption of iron, folate, vitamins, and minerals.

 The extraintestinal manifestations of CD in adults are more variable and may be the predominant presenting symptoms rather than the gastrointestinal manifestations, including hematological diseases such as iron deficiency anemia and hyposplenism; metabolic bone diseases like osteoporosis and osteomalacia,^10^ gastrointestinal diseases like inflammatory bowel disease and microscopic colitis,^11^ liver diseases like elevated liver enzymes, autoimmune hepatitis and primary biliary cholangitis,^12^ and kidney diseases like IgA nephropathy. Patients with CD may have other associated autoimmune disorders, such as type 1 diabetes mellitus^13^ and thyroid disease, including hypothyroidism more than hyperthyroidism.^14^

 Serological and histopathological findings that are compatible with CD are warranted. For patients with positive serologic testing, an upper endoscopy with small intestinal biopsy should be performed. Regarding serological evaluation, tTG IgA antibody is the single preferred test for the detection of CD in adults. In addition, total IgA levels should be measured concurrently. tTG IgA- and IgG-based tests are performed in patients with IgA deficiency.

 Patients with suspected CD should undergo upper endoscopy with small bowel biopsy to confirm the diagnosis. Endoscopic signs of CD include atrophic-appearing mucosa lacking folds, apparent fissures, nodularity, scalloping, and significant submucosal vascularity. However, endoscopic signs of CD have low sensitivity (59%–94%) and high specificity (92%-100%). These small bowel changes may be observed in various illnesses, such as giardiasis, autoimmune enteropathy, and HIV infection.^15^ Because of some heterogeneity in mucosal inflammation and injury, the degree of villous atrophy does not always correlate with the severity of clinical symptoms.

 The histological manifestations of celiac intestine range from moderate changes with merely an increase in intraepithelial lymphocytes to severely atrophic mucosa with complete loss of villi, increased epithelial apoptosis, and crypt hyperplasia. Using the Marsh-Oberhuber classification, intestinal lesions in CD were ranked according to their histological severity. Although not pathognomonic for CD, Marsh type 2 and 3 lesions aid in making the diagnosis.^16^

 Marsh proposed a four-stage grading system based on the dynamic development pattern of celiac lesions and the frequent discovery of CD cases with mild lesions, specifically, (1) type 1 infiltrative lesions, characterized by normal mucosal architecture with an increased number of intraepithelial lymphocytes (IELs), (2) type 2 hyperplastic lesions, characterized by an increase in crypt depth without villous flattening, (3) type 3 destructive lesions, characterized by villous atrophy and crypt hypertrophy; and (4) type 4 hypoplastic lesions, characterized by villous atrophy with normal crypt height and IEL count. Subsequently, a new standardized reporting approach based on the Marsh classification was developed, in which stage 3 was further divided into stages 3a, 3b, and 3c, characterized by mild villous flattening, substantial villous flattening, and complete villous atrophy.^17^

 Searching PubMed, looking for topics with NDCD in adults, revealed few papers with such a topic. This study tries to highlight the clinical features and investigations relevant to NDCD as well as clarify the important role of EGD in the diagnosis of NDCD.

## Materials and Methods

 This prospective study was conducted across multiple centers in five different cities in Iraq (Babylon, Basra, Kirkuk, Ninevah, and Salahalden). The study spanned a period of 2 years, from January 2023 to January 2025, and included 117 patients with NDCD from these five centers.

 A Google form was created to collect patients̓’ information. An ethical approval for the study was obtained from the ethical committee of the College of Medicine, University of Mosul, and informed consent was secured from all participants.

 All patients included in the study had no history of diarrhea and met the diagnostic criteria for CD, which was confirmed through serological and histopathological evaluation. In cases where there was a discrepancy between serology and pathology, HLA typing was used to confirm the diagnosis. Out of 143 patients whose data were initially collected, only 117 patients had full data and were included in the study.

 The data collected from patients included age, sex, residence, job, duration between symptom onset and diagnosis, main complaints, and associated symptoms such as bloating and gases, epigastric pain, nausea, weight loss, oral ulcers, constipation, loss of appetite, anemia, family history of CD, and other associated diseases, particularly autoimmune disorders.

 Weight, height and body mass index (BMI) were measured. Additional data included endoscopic findings, whether the diagnosis was settled after endoscopy, titers of anti-tTG (IgA and IgG), hemoglobin level, serum ferritin level, and the Marsh classification of duodenal biopsy.

 Esophagogastroduodenal endoscopy (EGD) was performed on all patients involved in the study, during which six biopsy samples were taken during EGD using biopsy forceps, four from the second part of the duodenum and two from the duodenal bulb. A histopathologist expert in gastrointestinal disorders reviewed duodenal biopsies, which were classified according to the Marsh-Oberhuber classification of CD in the duodenum.

 All patients included in the study exhibited histopathological features of CD, and most of them had elevated tTG-IgA levels (> 18 U/mL).

## Results

 The study included 117 patients, with 90 female (77%) and 27 male patients (23%), resulting in a female-to-male ratio of 3.3:1. The overall mean age of patients was 29 ± 13 years (29.42 ± 12.34 years for females and 26.11 ± 13.70 years for males). Notably, 94 patients (80%) were aged less than 40 years, and the youngest and oldest patients included in the study were aged 7 and 60 years, respectively.

 The mean age of the patients, according to the duration of the disease before the diagnosis, was as follows: 27.66 years ± 13.449 for those with < 1 year of illness, 31.27 years ± 11.570 for those between 1 and 2 years of illness, and 27.81 years ± 12.874 for those with ≥ 2 years of illness.

 Out of the 117 patients, 71 (60.7%) were married and resided in urban areas. Approximately half of the patients (58 patients, 49.6%) were symptomatic for 1–2 years before the diagnosis of CD, and a quarter of patients (30 patients, 25.6%) were symptomatic for 2 years or more.

 In terms of BMI), more than half of patients (66 patients, 54.4%) had normal BMI, over a quarter of patients (33 patients, 28.2%) were classified as overweight or obese, and only 18 patients (15.4%) were underweight (Table 1).

**Table 1:** Demographic characteristics of 117 patients

**Variables**		**Frequency**	**Percent**
Age groups (years)	11-20	39	33.3
	21-30	32	27.4
	31-40	23	19.7
	41-50	15	12.8
	51-60	8	6.8
Marital status	Married	71	60.7
	Single	46	39.3
Residency	Rural	46	39.3
	Urban	71	60.7
Duration of symptoms (years)	< 1	29	24.8
	1-2	58	49.6
	> 2	30	25.6
BMI (kg/m^2^)	Under weight	18	15.4
	Normal	66	56.4
	Overweight	20	17.1
	Obese	13	11.1

 The diagnosis of most cases (99 patients, 84.6%) of NDCD was made post-EGD, as doctors were familiar with the endoscopic findings associated with CD. The diagnosis was confirmed through duodenal biopsy and serology.

 The most common presenting symptom leading to EGD was epigastric pain, which was reported by 92 patients (78.6%). Other frequently associated symptoms include (in order of frequency)): gases and bloating (82%), weight loss (63.2%), loss of appetite (60.7%), oral ulcers (38.5%), constipation (35%), and nausea and vomiting (35%) (Table 2). Anemia was present in 101 patients (86.3%) with a mean Hb of 9.7 ± 1.9 mg/dL and a mean serum ferritin of 8.4 ± 5.7 ng/mL. Additionally, 73.5% of patients had low serum ferritin regardless of whether they were anemic or not, while 2% exhibited anemia with normal serum ferritin levels.

**Table 2:** Frequency of symptoms in patients with NDCD

**Variables**		**Frequency**	**Percentage**
Gas and bloating	No	21	17.9
	Yes	96	82.1
Nausea and vomiting	No	76	65.0
	Yes	41	35.0
Oral ulcer	No	72	61.5
	Yes	45	38.5
Loss of apatite	No	46	39.3
	Yes	71	60.7
Weight loss	No	43	36.8
	Yes	74	63.2
Anemia	No	16	13.7
	Yes	101	86.3
Epigastric pain	No	25	21.4
	Yes	92	78.6
Constipation	No	76	65.0
	Yes	41	35.0
Diagnosed after OGD	No	18	15.4
	Yes	99	84.6

**Figure 1:**
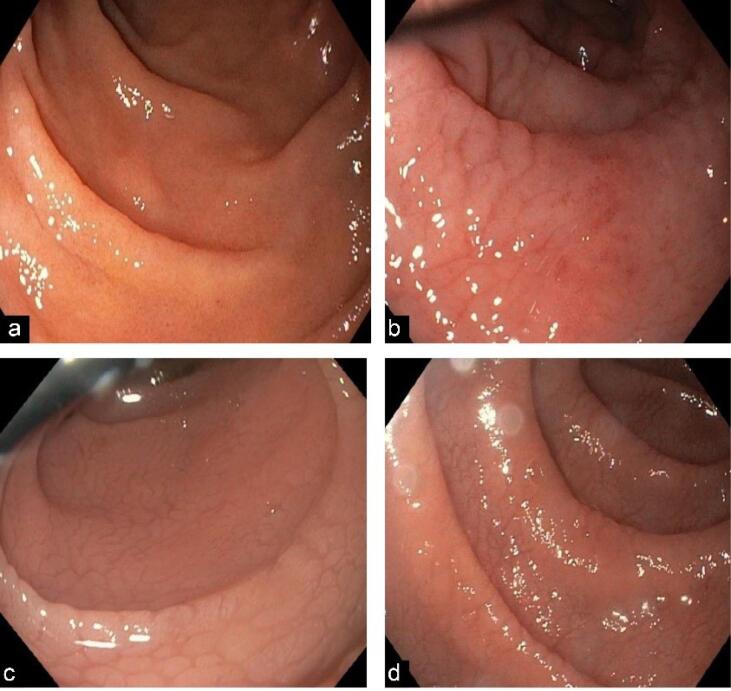
Appearance of duodenal mucosa during endoscopy; a: normal duodenal mucosa; b: scalloping of duodenal folds; c: mosaic and nodularity of duodenal folds; d: fissured duodenal folds

 Regarding the endoscopic characteristics of CD, 109 patients (94%) displayed an endoscopic feature in the form of nodularity (31 patients, 26.5%), scalloping (35 patients, 29.9%), and fissured (43 patients, 36.8%) duodenal folds. Only eight patients (6.8%) had normal endoscopy (Figure 1).

**Figure 2:**
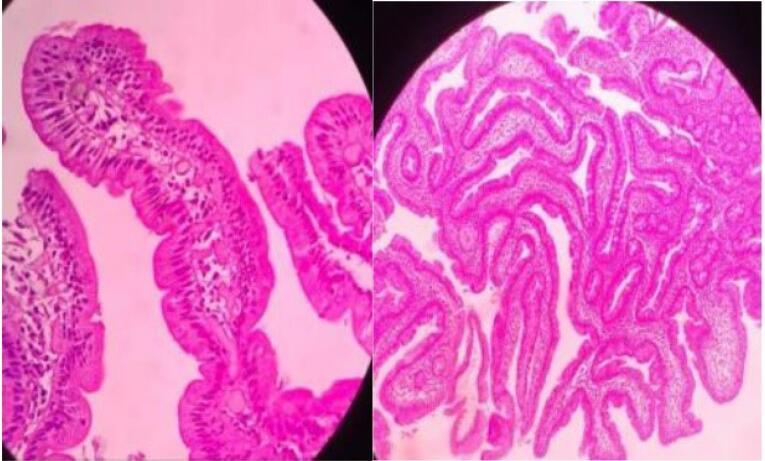
Normal duodenal mucosa with long villi and no increase in intraepithelial lymphocytes (power 40X)

**Figure 3:**
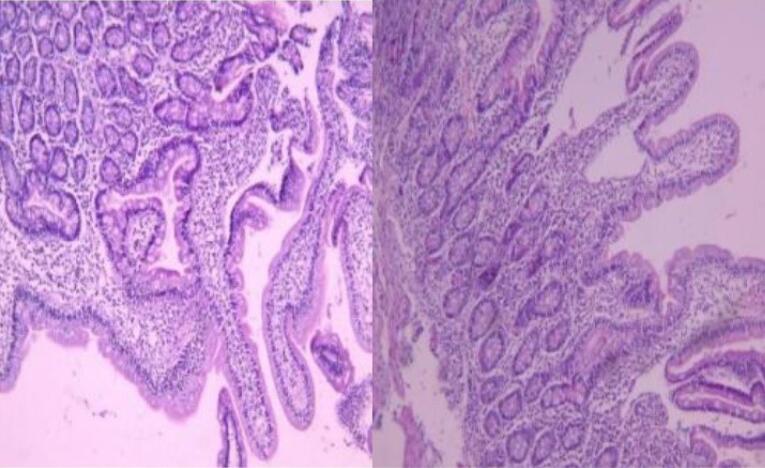
Increase intraepithelial lymphocytes with crypt hyperplasia of duodenal mucosa (power 40X)

**Figure 4:**
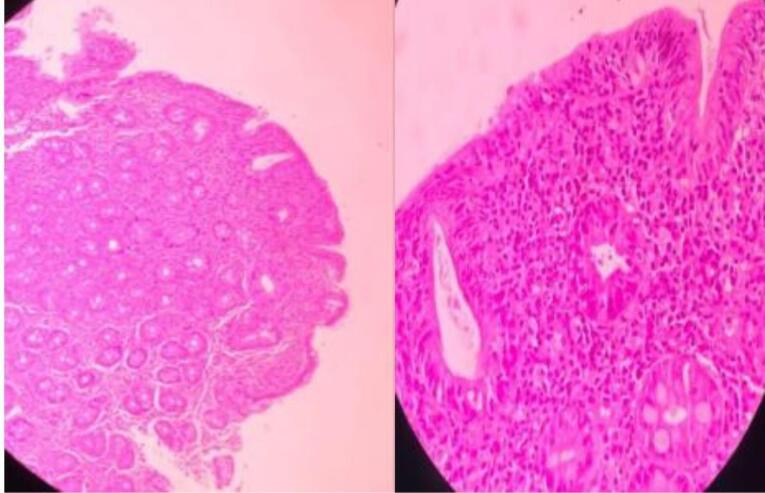
Total villous atrophy (Marsh 3) with a flat surface and increased intraepithelial lymphocytes of duodenal mucosa (power 40X)

 In the Marsh-Oberhuber classification of the duodenal biopsies, only one patient was classified as Marsh 1, 87 patients (75%) as Marsh 3, and 29 patients (24.8%) as Marsh 2 (Table 3, Figures 2, 3, and 4).

**Table 3:** Endoscopic findings of duodenal mucosa and Marsh classification

**Variables**		**Frequency**	**Percent**
Endoscopic finding of the duodenal mucosa	Fissured	43	36.8
	Nodularity	31	26.5
	Scalloping	35	29.9
	Normal	8	6.8
Marsh grades	Marsh 1	1	0.8
	Marsh 2	29	24.8
	Marsh 3a	25	21.4
	Marsh 3b	27	23.1
	Marsh 3c	35	29.9

 Furthermore, 43 patients (37%) reported a family history of CD among their first- and second-degree relatives. Concerning the association of CD with other diseases, six patients (5%) had type 1 (DM1), four patients (3.4%) had migraine, two patients (1.7%) had hypothyroidism, and one patient (0.8%) had ulcerative colitis.

## Discussion

 CD is more prevalent in women, and this study also found a higher incidence of NDCD in female patients, with a female-to-male ratio of 3.3:1. This aligns with other studies^18^ that reported a similar female-to-male ratio of 2.5 and 3.33, respectively, indicating that there is no difference in the female-to-male ratio in those with classical CD and NDCD.

 CD can manifest at any age; some studies have reported that CD has two peaks of onset: the first peak at 1-3 years of age and the second peak at 6-10 years,^19^ while other studies have suggested that the prevalence of CD in the elderly is comparable to that in patients less than 18 years of age.^20^ In our study, most patients (94 patients, 80%) were under 40 years old.

 Half of the patients included in this study were symptomatic for 1-2 years before the diagnosis of NDCD, while a quarter had symptoms for more than 2 years. This is consistent with previous studies that have noted delays in the diagnosis of CD and its associated symptoms.^21,22^

 There is no significant association between the age of patients with NDCD and the duration of disease prior to diagnosis (Table 4). This finding is consistent with other studies that reported a similar lack of correlation between age at diagnosis and duration of CD.^23^

**Table 4:** Association between the duration of NDCD and the age of patients at the time of diagnosis

			**Age**					***P*** ** value**
			**11-20**	**21-30**	**31-40**	**41-50**	**51-60**	
Duration (years)	< 1	No.	12	6	4	5	2	0.7
		%	30.8%	18.8%	17.4%	33.3%	25.0%	
	1-2	No.	21	17	10	6	4	
		%	53.8%	53.1%	43.5%	40.0%	50.0%	
	≥ 2	No.	6	9	9	4	2	
		%	15.4%	28.1%	39.1%	26.7%	25.0%	
Total		No.	No.	32	23	15	8	
		%	%	100.0%	100.0%	100.0%	100.0%	

 Contrary to the common belief that patients with CD are underweight, our study showed that only 18 patients (15.6%) were underweight. In fact, half of the patients had a normal BMI, and a quarter were classified as overweight. This is consistent with other studies that included patients with CD with different symptoms.^24^

 Compared with the general population, individuals with CD are more likely to experience dyspepsia, abdominal pain, bloating, and symptoms of gastroesophageal reflux. In our study, the most common presenting symptoms that led to endoscopy were epigastric pain, bloating and gases, weight loss, loss of appetite, oral ulcers, and constipation. Dyspepsia was more prevalent among our patients with NDCD (78.6%) compared with the general CD population, where only 30-40% reported dyspepsia. This suggests that patients with unexplained dyspepsia, particularly if associated with other symptoms such as bloating and weight loss, should undergo duodenal biopsies.^25^

 Iron deficiency anemia (IDA) was present in most patients with NDCD (86.3%), which indicates a higher prevalence of IDA in NDCD compared with other studies that found around half of the patients with CD had IDA.^26,27^ This increased prevalence of IDA among patients with NDCD is probably because unexplained IDA is considered a common reason to look for CD, and IDA was a common indication for EGD and small bowel biopsy in our cohort.

 There was no relationship between the duration of CD before diagnosis and anti-tTG levels (Table 5).

**Table 5:** Relation between the duration of NDCD and anti-tTG level

		**N**	**Mean Anti-tTG**	**Standard deviation**	***P*** ** value **
Duration (years)	< 1	29	96.90	40.96	0.4
	1-2	30	58.66	14.20	
	> 2	58	57.13	13.64	

 Endoscopic changes in the duodenal mucosa of patients with CD have low sensitivity but high specificity.^28^ In our study, only eight patients (6.8%) had normal endoscopy findings, while 109 patients (94%) had endoscopic changes compatible with mucosal atrophy. A review of the literature indicates that there is no clear consensus on which endoscopic findings are more sensitive or specific for CD, with the sensitivity of the endoscopic changes varying from 50% to 90%.^29^ Most of NDCD cases (99 patients, 84.6%) included in this study were diagnosed following EGD due to specific symptoms. This highlights the role of endoscopy in identifying mucosal atrophy changes in patients with CD, particularly when performed by experienced endoscopists, along with subsequent duodenal biopsy.^28^

 Among the patients studied, only one had Marsh-Oberhuber grade 1, while 75% (87 patients) had Marsh grade 3, and 24.8% (29 patients) had Marsh grade 2. There was no significant association between the endoscopic findings and the Marsh classification, as shown in Table 6, which is consistent with the findings of other studies conducted on patients with CD.^30^

**Table 6:** Association between Marsh-Oberhuber grade and endoscopic findings

			**Marsh 1**	**Marsh 2**	**Marsh 3a**	**Marsh 3b**	**Marsh 3c**	***P*** ** value**
Endoscopic finding	Fissured	No.	1	12	4	11	15	0.5
		%	100.0%	41.4%	16.0%	40.7%	42.9%	
	Nodularity	No.	0	6	11	6	8	
		%	0.0%	20.7%	44.0%	22.2%	22.9%	
	Normal	No.	0	1	2	3	2	
		%	0.0%	3.4%	8.0%	11.1%	5.7%	
	Scalloping	No.	0	10	8	7	10	
		%	0.0%	34.5%	32.0%	25.9%	28.6%	
Total		No.	1	29	25	27	35	
		%	100.0%	100.0%	100.0%	100.0%	100.0%	

## Conclusion

 NDCD is common among young females, but the diagnosis is often delayed for more than a year. This delay may be due to a lack of classical gastrointestinal symptoms. Most patients with NDCD were of normal or overweight. Dyspepsia and IDA were more prevalent in NDCD compared with classical CD. Although endoscopy was considered a useful tool to diagnose NDCD, there was no correlation between the endoscopic findings and the duration of the disease.
